# The Impact of Chemical-Mechanical Ex Situ Aging on PFSA Membranes for Fuel Cells

**DOI:** 10.3390/membranes11050366

**Published:** 2021-05-18

**Authors:** Mylène Robert, Assma El Kaddouri, Jean-Christophe Perrin, Kévin Mozet, Jérôme Dillet, Jean-Yves Morel, Olivier Lottin

**Affiliations:** Université de Lorraine, CNRS, LEMTA, F-54000 Nancy, France; assma.el-kaddouri@univ-lorraine.fr (A.E.K.); jean-christophe.perrin@univ-lorraine.fr (J.-C.P.); kevin.mozet@univ-lorraine.fr (K.M.); jerome.dillet@univ-lorraine.fr (J.D.); jean-yves.morel@univ-lorraine.fr (J.-Y.M.); olivier.lottin@univ-lorraine.fr (O.L.)

**Keywords:** chemical degradation, durability, mechanical fatigue, Nafion^™^ membranes, PEM fuel cell

## Abstract

A proton-exchange membrane fuel cell (PEMFC) constitutes today one of the preferred technologies to promote hydrogen-based alternative energies. However, the large-scale deployment of PEMFCs is still hampered by insufficient durability and reliability. In particular, the degradation of the polyelectrolyte membrane, caused by harsh mechanical and chemical stresses experienced during fuel cell operation, has been identified as one of the main factors restricting the PEMFC lifetime. An innovative chemical-mechanical ex situ aging device was developed to simultaneously expose the membrane to mechanical fatigue and an oxidizing environment (i.e., free radicals) in order to reproduce conditions close to those encountered in fuel cell systems. A cyclic compressive stress of 5 or 10 MPa was applied during several hours while a degrading solution (H_2_O_2_ or a Fenton solution) was circulated in contact with the membrane. The results demonstrated that both composite Nafion^™^ XL and non-reinforced Nafion^™^ NR211 membranes are significantly degraded by the conjoint mechanical and chemical stress exposure. The fluoride emission rate (FER) was generally slightly lower with XL than with NR211, which could be attributed to the degradation mitigation strategies developed for composite XL, except when the pressure level or the aging duration were increased, suggesting a limitation of the improved durability of XL.

## 1. Introduction

Proton Exchange Membrane Fuel Cells (PEMFCs) are promising clean electricity generators with numerous applications in stationary and transport domains. However, even though hydrogen is recognized as an efficient and safe alternative to fossil fuels, the wide-range commercialization of fuel cells is still hampered by high manufacturing costs and a restricted lifetime. Numerous studies conducted in recent decades have already provided key understandings about the aging phenomena and degradation mechanisms of PEMFC materials, among which is the polyelectrolyte membrane, a crucial component for their operation [1,2,3,4]. In these studies, it has been clearly demonstrated that the membrane was exposed to harsh conditions during operation, involving significant chemical and mechanical stresses that could lead to severe degradation of the membrane structure and properties and, in the worst case, to fuel cell shutdown due to the membrane failure. In the last few years, the incorporation of inert mechanical reinforcements and/or chemical stabilizers has made it possible to reduce the thickness and the ionic resistivity of membranes without compromising their mechanical robustness and chemical stability [5].

Nowadays, perfluorosulfonic acid (PFSA) membranes are the most commonly used in PEMFCs due to their remarkable proton conductivity and chemical-mechanical stability. Nonetheless, they still suffer from a high degradation rate limiting the fuel cell lifetime [5]. Membranes indeed experience harsh conditions in fuel cell operation such as an aggressive chemical environment and important mechanical stress. 

This aggressive environment results mostly from the formation of hydrogen peroxide (H_2_O_2_) and its subsequent decomposition into highly reactive oxygen species (hydrogen H^•^, hydroxyl HO^•^ and hydroperoxyl HOO^•^ radicals) [5,6,7]. The radicals can then attack the most vulnerable sites of the PFSA, leading to the scission of polymer chains (backbone and/or side chains) [5] and thus to the membrane thinning [8,9]. On the other hand, the humidity cycling and the stack clamping pressure exerted on the membrane during fuel cell operation cause important variations in the membrane water content, thus generating a mechanical fatigue that weakens its structure and can lead to the formation and growth of cracks and pinholes [10,11,12]. These days, it is well accepted that the membrane decomposition results from the synergistic interaction between the mechanical fatigue and the chemical degradation [1,2,13,14,15].

The Fenton reaction is a widely–used ex–situ accelerated stress test (AST) enabling the reproduction of an oxidizing environment close to that observed during fuel cell operation [16]. It consists of reacting hydrogen peroxide (H_2_O_2_) with iron ions (Fe^2+^/Fe^3+^) to form hydroxyl HO^•^ and hydroperoxyl HOO^•^ radicals with an accelerated way to rapidly evaluate the durability of PFSA membranes against chemical degradations. However, more complete and elaborate ex situ protocols are required to also consider the mechanical stress exerted on the membrane in fuel cells.

In recent years, mitigation strategies have been developed to enhance the durability and performances of PFSA membranes. For instance, DuPont de Nemours^™^ and W. L. Gore & Associate Inc. have manufactured mechanically reinforced membranes by introducing a thin microporous layer of polytetrafluoroethylene (PTFE): the Nafion^™^ XL and Gore-SELECT^®^ membranes, respectively. This process enables the elaboration of thinner membranes with a better mechanical endurance while their proton conductivity remains comparable with their unreinforced analogue [17,18]. Furthermore, it has been demonstrated that the introduction of radical scavengers based on cerium species efficiently reduced the PFSA decomposition as a result of the radical attacks [19,20,21]. However, despite the presence of mechanical reinforcement and/or radical scavengers, the lifetime of composite membranes is still limited for long-term fuel cell operation [22,23]. In addition, it is worth noting that even though mechanical reinforcement and radical scavengers are the most frequently used mitigation strategies to increase the durability of PFSA membranes and thus the PEMFC lifetime, other solutions have been proposed in the literature, for instance, new PFSA ionomers with structural features as well as semi-interpenetrating polymer networks have been recently developed to enhance membrane properties [24,25] while a few authors improved the efficiency of the catalyst layers by using a highly dense, well-ordered and cone-shaped Nafion^™^ array [26].

Nevertheless, among innumerable studies focusing independently on chemical or mechanical degradations these past decades, only a few have considered the effect of combined stresses on PFSA membranes [1,2,13,14,15]. The present study therefore aims to study the impact of a conjoint chemical-mechanical stress on composite and non-reinforced PFSA membranes. For that purpose, a specific home-made device was elaborated in order to couple mechanical and chemical stress exposure in conditions believed to reproduce fuel cell operation.

## 2. Materials and Methods

### 2.1. Material and Preparation

Two commercial membranes were investigated in this study: the composite Nafion™ XL and the non-reinforced Nafion™ NR211 membranes. They have similar chemical compositions based on perfluorosulfonic acid (PFSA), similar ion-exchange capacities (IEC)—0.92 meq/g for XL and 0.98 meq/g for NR211—as well as similar thicknesses of about 25–30 µm. Nevertheless, unlike NR211, the XL membrane contains an additional PTFE-rich reinforcement and cerium-based radical scavengers for improved mechanical and chemical endurance. These two membranes were compared to study the contribution of such mitigation strategies with coupled mechanical and chemical degradations. Finally, the size of the membrane samples used for the various chemical-mechanical aging tests was approximately 40–45 mm wide and 70–75 mm long.

Prior to aging tests or measurements, the commercial membrane samples were pretreated according to a standard procedure close to those already been established by Xu et al. [27].

### 2.2. Experimental Setup and Aging Tests Parameters

**Figure 1:**
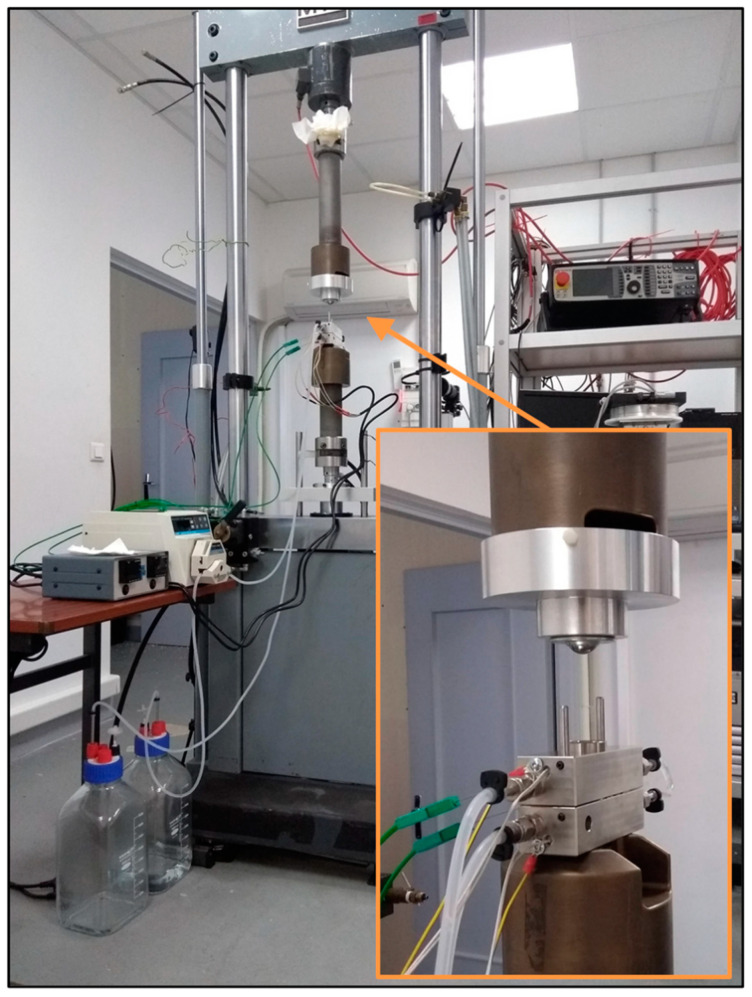
Photograph of the chemical-mechanical aging device. The inset focuses on the aging cell and the adapter specifically designed for this study.

A specific custom-made device was elaborated in order to reproduce operating conditions close to those exerted on PFSA membranes in fuel cell systems and thus study the impact of conjoint chemical and mechanical stress on the membrane [2]. The chemical-mechanical aging experiments consisted of applying a compressive stress on the membrane while exposed to an oxidizing environment. In this regard, an aging cell composed of two symmetric half-cells made in 316L stainless steel was machined and a single serpentine flow channel like those of fuel cell flow field plates was engraved on their surface. The lands and channels had a width of 1 mm and a depth of 0.7 mm (Figure 1). The effective area where the membrane was exposed to both mechanical and chemical stresses was approximately 19.5 × 39.5 mm² per half-cell.

During the aging tests, the membrane was sandwiched between the two half–cells and the aging cell was then inserted between the clamp of an electromechanical universal testing machine (MTS load frame model 312.21,MTS Systems, Créteil, France). In addition, the aging cell was heated to 80 °C thanks to heater cartridges inserted into each half-cell close to the membrane and an EPDM (ethylene propylene diene monomer) O-ring gasket was used to guarantee perfect sealing. Prior to each aging test, it was necessary to determine the minimal pressure required to compress the gasket and thus ensure a perfect sealing. This minimal pressure corresponded in this study with the 0 MPa reference pressure level.

The chemical stress was induced by circulating a continuous flow of degrading solution (H_2_O_2_ or Fenton solution) through the flow channel of the aging cell with a flow rate of 3.0 mL/min, which corresponded with a residence time of the solution in contact with the membrane of about 10 s per half–cell. Based on the literature, mild conditions were replicated by using a 3 vol % H_2_O_2_ solution while a Fenton solution containing 3 vol % of H_2_O_2_ and 1 ppm of ferrous ions Fe^2+^ was circulated to provoke more aggressive conditions [2,28].

A mechanical fatigue was applied on the membrane through the channel ribs in order to reproduce on the one hand the swelling/shrinkage cycles (i.e., by applying a cyclic compressive stress) or, on the other hand, the stack clamping pressure (i.e., by maintaining a static compressive stress) experienced by the membrane during fuel cell operation. The cyclic compressive stress consisted in a sinusoidal profile oscillating between 0 and 5 or 10 MPa [1,29] with a frequency of 0.1 Hz while the static compressive stress was maintained at constant pressure level. 

According to these operating conditions, one hour of a conjoint mechanical-chemical aging test could be considered as roughly equivalent to one year of daily operation with one startup/shutdown per day (i.e., 360 swelling/shrinkage cycles). Table 1 summarizes the main operating conditions in which the various chemical-mechanical aging tests were performed.

After each test, the membrane samples were extracted from the cell, immersed in distilled water and treated to eliminate cationic contaminants. For that purpose, the samples were soaked in a complexing solution of EDTA-Na_2_ (0.01 mol/L) at room temperature overnight before being boiled in a nitric acid HNO_3_ solution (1 mol/L) at 80 °C for 2 h and rinsed in distilled water at 80 °C, again for 2 h. Finally, the samples were dried in an oven at 60 °C for 20 h before being analyzed. The solutions that circulated through the aging cell were collected and stored for (possible) further analysis.

### 2.3. Quantification of the Fluoride Emission Rates 

The emission of fluoride ions, one of the most monitored PFSA degradation products, is frequently considered as a reliable indicator of the PFSA chemical degradation and can be easily assessed using, in our case, a fluoride-ion selective electrode (DX219, Mettler Toledo, Viroflay, France). The electrode was calibrated over the 0.057–19 ppm range using various calibration solutions prepared from a commercial standard solution (ISE standard F 1000 ppm, Mettler Toledo, Viroflay, France). The fluoride emission rate (FER) is expressed here in mg of fluoride ions released per gram of dry membrane per hour (mg/g_Nafion_/h).

## 3. Results

### 3.1. Impact of a Cyclic Compressive Stress

**Figure 2:**
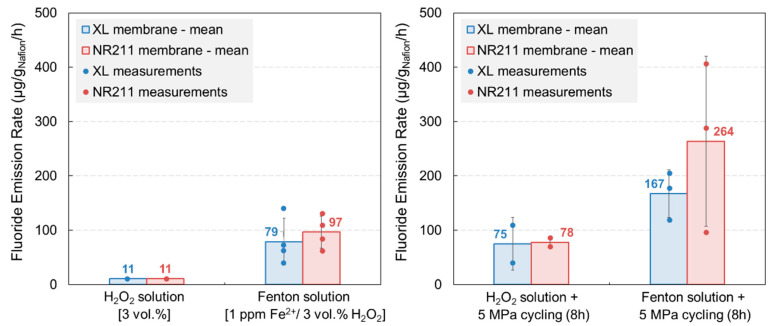
Fluoride emission rates (FER) of Nafion™ XL and NR211 membranes after chemical aging (to the left) and after chemical-mechanical aging (to the right).

First, accelerated stress tests coupling mechanical and chemical stresses were performed by applying a cyclic compressive stress to mimic the swelling/shrinkage sequences generated by the water content changes of the membrane during a transient fuel cell operation. A 5 MPa pressure was exerted on the membrane while a degrading solution was circulated through the aging cell for 8 h: a 3 vol % H_2_O_2_ solution for mild conditions and a Fenton solution with an optimal concentration of Fenton’s reagents for aggressive conditions [2,28]. The fluoride emission rates (FER) were evaluated by analyzing the circulating solutions collected at the end of each aging test and compared with those measured for pure chemical stress tests to estimate the impact of mechanical fatigue on the polymer chemical decomposition (Figure 2).

As observed in the case of ex situ chemical stress tests, the degradation rate obtained after mechanical-chemical stress tests for both XL and NR211 membranes was higher with a Fenton solution exposure than with the H_2_O_2_ solution exposure, which indicated that the mechanical fatigue did not predominate over the chemical degradation, i.e., shifting from hydrogen peroxide to the Fenton solution had a higher impact than adding a mechanical stress. Nonetheless, coupling mechanical and chemical stresses accelerated the membrane degradation because a FER twice to thrice higher was obtained for both membranes by applying a 5 MPa compression while circulating a Fenton solution instead of exposing membranes to the Fenton solution only. Furthermore, the XL membrane was significantly degraded by the conjoint chemical and mechanical stress exposure despite the presence of an additional mechanical reinforcement and radical scavengers in comparison with its non-reinforced analogue, the NR211 membrane.

Supplementary experiments were then carried out by increasing the pressure level from 5 to 10 MPa and the aging duration from 8 to 20 h to evaluate the degradation behavior of XL and NR211 membranes in more severe conditions (Table 2). These new tests were carried out using Fenton solutions only.

The degradation rate of both membranes increased significantly when the pressure level was doubled: the FER of XL was almost twice higher while a slight increase of 22% was noticed for NR211. Furthermore, by extending the aging duration from 8 to 20 h, the FER was considerably amplified for both XL and NR211 membranes, indicating an acceleration of the degradation. Moreover, when more severe operating conditions were applied on the membranes, the results showed that both membranes were degraded in equal proportion, which could suggest that the mitigation strategies to improve XL durability were insufficient to effectively prevent the chemical decomposition of the polymer at a higher pressure level or after long-term aging. It is thus possible that the PTFE–based reinforcement layer and/or the cerium-based radical scavengers were no longer able to inhibit the chemical decomposition of the polymer when the mechanical fatigue became too important, thus leading to a degradation behavior through fluoride emission rates similar to that of the non-reinforced NR211 membrane. However, in the light of our current results, it cannot be concluded definitely if one strategy prevailed over the other or whether both were involved in preventing the membrane from degradation.

### 3.2. Impact of a Static Compressive Stress

**Figure 3:**
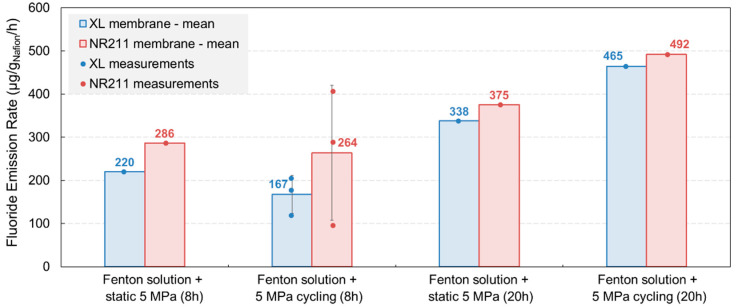
Comparison of the FER evolution after conjoint mechanical and chemical stress tests with static compression or cyclic compression for XL and NR211 membranes.

In a second phase, a static compressive stress was applied to mimic the stack clamping pressure exerted on membranes during fuel cell operation. In that purpose, two aging tests similar to those previously presented were carried out by maintaining a constant pressure level of 5 MPa on membranes while circulating the Fenton solution during 8 or 20 h. Figure 3 illustrates the evolution of the FER after these two static aging tests for both XL and NR211 membranes in comparison with the results obtained in the case of cyclic aging tests. Similarly, an acceleration of the membrane decomposition was observed by extending the aging duration as a FER increase of about 30 % and 50 % for XL and NR211 membranes, respectively, after combined static compressive stress and Fenton solution exposure. In addition, and as previously observed for cyclic compression exposure, both membranes were degraded in a very similar proportion when the aging duration was increased from 8 to 20 h, which could indicate that maintaining a constant pressure level on the XL membrane also restricted its improved durability.

Furthermore, the application of a static 5 MPa compression seemed slightly more aggressive than that of a cyclic 5 MPa compression for both membranes in the case of the short-term aging tests (8 h). However, this trend was not observed after long-term aging (20 h), which could indicate that, at an equal pressure level, applying a quite severe cyclic compressive stress was more detrimental for Nafion^™^ membranes than maintaining a constant compression.

## 4. Conclusions

The degradation behavior of both composite Nafion^™^ XL and non-reinforced Nafion^™^ NR211 membranes was investigated after various chemical-mechanical aging tests. In this regard, an innovative device able to couple mechanical fatigue and an aggressive chemical environment exposure to the membrane was elaborated in order to mimic fuel cell operating conditions with an ex situ approach. It is therefore required to develop new accelerated stress tests coupling mechanical and chemical stresses to evaluate efficiently and rapidly the durability of PFSA membranes in conditions close to those experienced during fuel cell operation. This study is believed to offer new understandings about the impact of conjoint mechanical and chemical stresses on the degradation of Nafion^™^ membranes.

The first results demonstrated that the degradation behavior of both XL and NR211 membranes was not modified when chemical stress was coupled to a mechanical stress: the FER remained higher by exposing the membrane to the Fenton solution instead of the H_2_O_2_ solution only. However, adding a mechanical fatigue to the chemical stress significantly accelerated the polymer decomposition. 

The comparison of XL and NR211 degradation behaviors seemed to indicate that XL was slightly more stable than NR211 against conjoint chemical and mechanical stresses. This discrepancy could be attributed to the presence of an additional PTFE-based reinforcement providing a better mechanical strength and dimensional stability to the membrane and/or to the presence of radical scavengers preventing the polymer decomposition against radical attacks. Nevertheless, the mitigation strategies developed for XL did not prevent the polymer decomposition after the application of severe mechanical solicitations (i.e., long-term aging or a high pressure level) because the degradation rates of XL were comparable with those obtained with the non-reinforced NR211. 

The custom-made device and the associated accelerated aging protocols presented in this study constitute a first step towards larger investigations to provide new insights about the chemical-mechanical aging of PFSA membranes and opens up various perspectives. For instance, introducing gas diffusion layers (GDL) between the flow field plates and the membrane in our aging cell or modifying the operating procedure to expose the membrane to a gaseous H_2_O_2_ environment instead of an aqueous solution could better mimic the mechanical constraint exerted on membranes during fuel cell operation. Finally, one can also imagine lengthening the aging duration to more than a few tens of hours to further test the membrane durability.

## Figures and Tables

**Table 1:** Summary of the operating conditions used for the ex situ mechanical-chemical aging tests.

Mechanical Strength	Chemical Conditions	Duration	Number of Tests
5 MPa cycling	H_2_O_2_ solution	8 h	2
5 MPa cycling	Fenton solution	8 h	3
Static 5 MPa	Fenton solution	8 h	1
10 MPa cycling	Fenton solution	8 h	2
5 MPa cycling	Fenton solution	20 h	1
Static 5 MPa	Fenton solution	20 h	1

**Table 2:** Evolution of the fluoride emission rate as a function of the severity of conjoint mechanical and chemical stress exerted on Nafion^™^ XL and NR211 membranes. Part of the data presented here has been already published in a previous work [2] but the values have been corrected here by taking into account the parasitic contribution of the TISAB II solution used for the measurements.

FER (µg/g_Nafion_/h)	Cyclic 5 MPa + Fenton Solution (8 h)	Cyclic 10 MPa + Fenton Solution (8 h)	Cyclic 5 MPa + Fenton Solution (20 h)
XL membrane	167 ± 44	316 ± 44	465
NR211 membrane	264 ± 157	324 ± 79	492

## Data Availability

Not applicable.
